# Specific motor cortex hypoexcitability and hypoactivation in COPD patients with peripheral muscle weakness

**DOI:** 10.1186/s12890-019-1042-0

**Published:** 2020-01-03

**Authors:** Francois Alexandre, Nelly Héraud, Emilie Tremey, Nicolas Oliver, Dominique Bourgouin, Alain Varray

**Affiliations:** 1 Les Cliniques du Souffle, Research Department, Groupe 5 Santé, 800 avenue Joseph Vallot, 34700 Lodève, France; 2 Euromov Laboratory, University of Montpellier, Montpellier, France

**Keywords:** Chronic obstructive pulmonary disease, Motor cortex, Peripheral muscle weakness, Corticospinal excitability

## Abstract

**Background:**

Peripheral muscle weakness can be caused by both peripheral muscle and neural alterations. Although peripheral alterations cannot totally explain peripheral muscle weakness in COPD, the existence of an activation deficit remains controversial. The heterogeneity of muscle weakness (between 32 and 57% of COPD patients) is generally not controlled in studies and could explain this discrepancy. This study aimed to specifically compare voluntary and stimulated activation levels in COPD patients with and without muscle weakness.

**Methods:**

Twenty-two patients with quadriceps weakness (COPD_MW_), 18 patients with preserved quadriceps strength (COPD_NoMW_) and 20 controls were recruited. Voluntary activation was measured through peripheral nerve (VA_peripheral_) and transcranial magnetic (VA_cortical_) stimulation. Corticospinal and spinal excitability (MEP/Mmax and Hmax/Mmax) and corticospinal inhibition (silent period duration) were assessed during maximal voluntary quadriceps contractions.

**Results:**

COPD_MW_ exhibited lower VA_cortical_ and lower MEP/Mmax compared with COPD_NoMW_ (*p* < 0.05). Hmax/Mmax was not significantly different between groups (*p* = 0.25). Silent period duration was longer in the two groups of COPD patients compared with controls (*p* < 0.01). Interestingly, there were no significant differences between all COPD patients taken together and controls regarding VA_cortical_ and MEP/Mmax.

**Conclusions:**

COPD patients with muscle weakness have reduced voluntary activation without altered spinal excitability. Corticospinal inhibition is higher in COPD regardless of muscle weakness. Therefore, reduced cortical excitability and a voluntary activation deficit from the motor cortex are the most likely cortical mechanisms implicated in COPD muscle weakness. The mechanisms responsible for cortical impairment and possible therapeutic interventions need to be addressed.

## Background

Central (respiratory) and peripheral (limb) muscle weakness is one of the main systemic effects of chronic obstructive pulmonary disease (COPD) [1]. It primarily affects the lower limb muscles [2], contributes to exercise intolerance [3] and is associated with increasing disability and mortality [4, 5].

Peripheral muscle weakness can be caused by both peripheral (muscle mass and contractile properties) and neural alterations [6, 7]. Several studies have indicated that peripheral mechanisms do not account for all the strength loss in COPD. For example, lower quadriceps strength per unit of muscle cross-sectional area was found despite normal contractile properties in COPD patients [8]. In addition, lower quadriceps strength is not observed in some patients when peripheral nerve stimulation is used instead of voluntary contraction [9, 10]. This raises the hypothesis of altered neural drive to the muscle in COPD.

Surprisingly, the validation of this hypothesis remains quite controversial. By using the twitch interpolation method, most studies have failed to observe an activation deficit in COPD during maximal voluntary contractions [11–13], while others have clearly shown this deficit during submaximal voluntary contractions [14]. By using a different method and applying sensors directly to the scalp, lower neural drive from the motor cortex was reported during maximal and submaximal voluntary contractions in COPD [15]. Both methodological concerns and the heterogeneity of muscle weakness in COPD could explain these discrepancies. First, the twitch interpolation method may be biased during maximal voluntary contractions, as the relationship between voluntary strength and the twitch-like increment in strength is no longer linear at high intensities [16–18]. The measurement of voluntary activation by motor cortex stimulation could help to resolve this issue [19]. Second, it is well known that muscle weakness is not ever-present in COPD patients since its prevalence is between 32 and 57% [20, 21]. None of the aforementioned studies discriminated the patients with and without muscle weakness. Thus, different proportions of COPD patients with versus without muscle weakness, depending on the study, could have biased the overall results.

Furthermore, lower neural drive to the muscle can be ascribed to at least three mechanisms, which have never been questioned in the context of muscle weakness in COPD. First, decreased excitation from the brain is strongly expected in these patients given the decreased gray matter density in the motor and prefrontal cortex [22, 23] and the presence of white matter lesions in the pyramidal neurons [24]. Second, lengthened latency and lower amplitude of the maximal compound muscle action potential (Mmax) have also been described in COPD patients and suggest impaired neuromuscular transmission at the motor neuron and/or the motor plate level [25–28]. Third, higher supraspinal inhibition during voluntary contraction could contribute to lowering the motor output by inhibiting the neural drive in COPD. Indeed, inflammatory mediators as well as lactate and protons [29] induce increased activity in group III or IV muscle afferents, which acts supraspinally to limit motor cortical output [30]. As COPD patients are known to exhibit high levels of chronic systemic inflammation [31] and a predominance of type II glycolytic fibers leading to elevated muscle glycolytic activity [32], the hypothesis of a motor output decrease due to increased inhibition is relevant.

The aim of the study was to address the question of a specific activation deficit in COPD with muscle weakness, and if so, by which mechanisms. We hypothesized lower motor cortex activation and excitability, higher corticospinal inhibition, and lower Mmax amplitude in patients with COPD and muscle weakness, as compared with COPD patients with normal muscle strength.

## Methods

### Participants

**Fig. 1:**
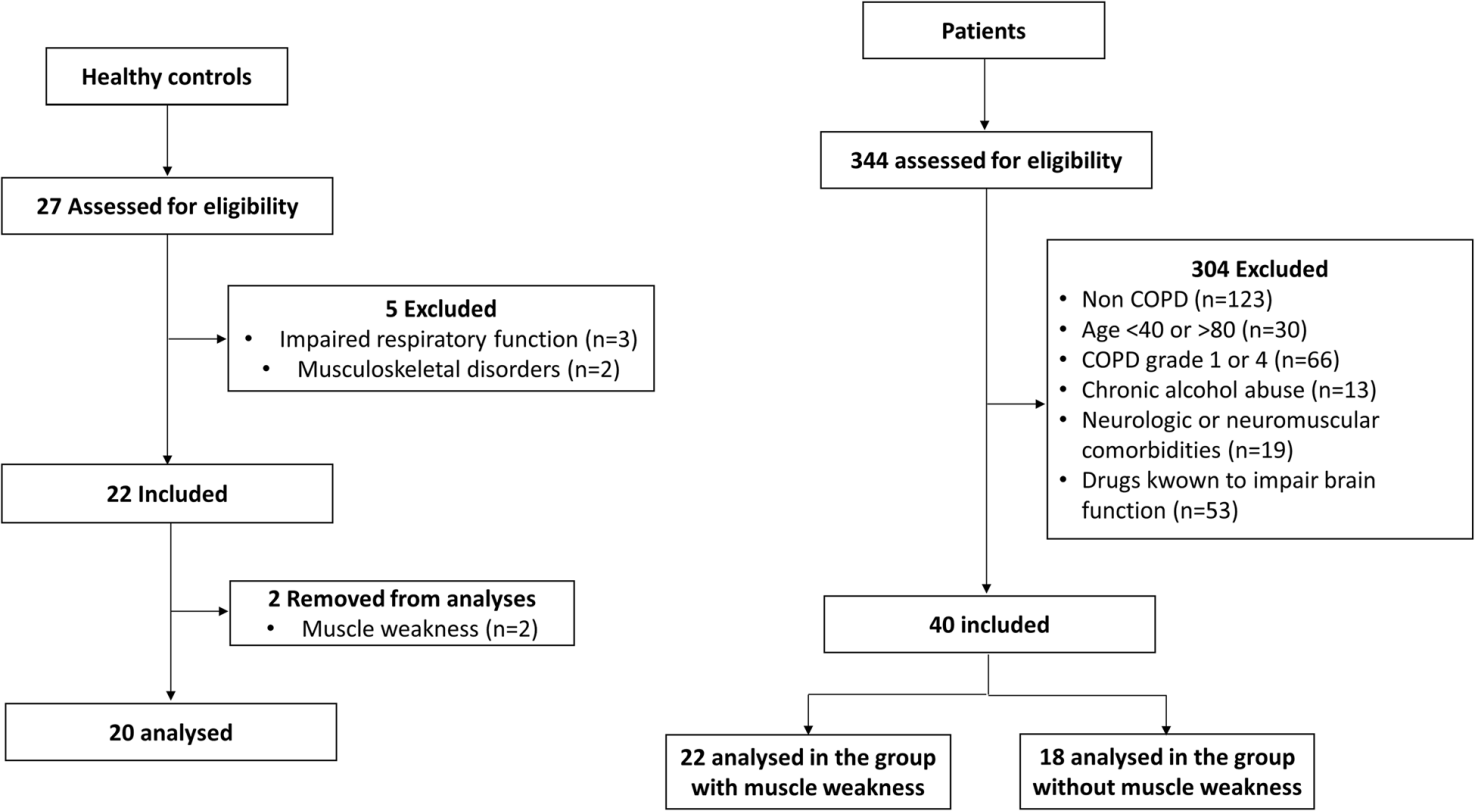
Flow diagram of the study

Forty COPD patients and 22 healthy controls, aged between 40 and 80 years, were recruited for the study (Fig. 1). The COPD patients were recruited and tested at their entrance in two French pulmonary rehabilitation centers (Cliniques du Souffle La Vallonie, Lodève, and Les Clarines, Riom-ès-Montagne, France) between 2012 and 2014. The healthy controls were recruited through an ad in a local newspaper within the same period. The participation criteria for the COPD patients were a diagnosis of COPD with forced expiratory volume in the 1st second (FEV_1_) between 30 and 80% of the theoretical values (GOLD 2 and 3), with no exacerbation or weight loss in the month preceding the study. The non-inclusion criteria were the same for patients and controls: inability to give written consent, inability to perform the experimental maneuvers, impaired visual function, use of drugs known to impact brain function (GABA agonist, Z-drugs, tricyclic antidepressants, melatoninergic antidepressants, selective serotonin/noradrenalin reuptake inhibitors and opioid receptor agonists), chronic current or past alcohol abuse (> 14 units of alcohol per week), mental disorder and neurologic or neuromuscular disease. For the diagnosis of peripheral muscle weakness, the isometric maximal quadriceps torque (QMVC) of each participant was expressed as a percentage of predicted values obtained from the national isometric muscle strength database consortium [33]. Patients with QMVC below 80% of predicted values were then assigned to the muscle weakness group (COPD_MW_) and the others to the non-muscle weakness group (COPD_NoMW_) [34]. Healthy controls with peripheral muscle weakness were excluded from the analyses (*n* = 2). All participants gave written consent. Procedures were approved by the local ethics committee (CPP Sud-Est VI, Clermont-Ferrand, number AU980) and complied with the principles of the Declaration of Helsinki for human experimentation.

### Pulmonary function tests

#### Plethysmography

Both patients and controls underwent plethysmography (V6200 Autobox, Sensormedics Corp., Yorba Linda, CA, USA). Measurements included forced vital capacity (FVC) and FEV_1_. The presence of persistent airflow obstruction and thus COPD was defined by a postbronchodilatator FEV_1_/FVC ratio < 0.7 [35]. The FEV_1_ values were expressed as a percentage of predicted value [36].

#### Blood gas analyses

Measurement of blood gases (PaO_2_ and PaCO_2_) collected from the radial artery was performed in resting patients while they breathed room air, using a blood gas analyzer (ABL 825, Radiometer Medical, Bronshoj, Denmark).

### Neuromuscular tests

#### Experimental design

After determination of the dominant leg [37], the participants were comfortably seated on a dedicated ergometer for knee extensor testing (Quadriergoforme, Aleo Industrie, Salome, France) equipped with a strain gauge torque sensor (Captels, Saint Mathieu de Treviers, France). The hip and the knee angle were set at 90°. The pelvis and the proximal extremity of the patella were securely attached to the chair in order to minimize movements of adjacent muscles. All the experimental manoeuvers of the protocol were done on the ergometer and in the same body position (including stimulations at rest). The participants were systematically familiarized to the experimental procedures the day before the protocol through a physical training session. This session included transcranial magnetic and femoral nerve stimulation recruitment curves, followed by 3 maximal voluntary contractions and several submaximal voluntary contractions at 30 and 50% of MVC lasting 5 s or until the targets were correctly reached, with superimposed transcranial magnetic and femoral nerve stimulations.

#### Evaluation of isometric maximal quadriceps torque

Isometric maximal quadriceps torque of the dominant leg was assessed as the highest torque value recorded during the protocol. Participants were verbally encouraged during each contraction to ensure maximal personal implication. QMVC was expressed in Nm and as a percentage of predicted values obtained from the national isometric muscle strength database consortium [33]. The maximal electrically evoked torque (quadriceps peak twitch, QPt) was assessed at rest as the highest twitch response induced by I_Mmax_ femoral nerve stimulation (I_Mmax_ determination is described in the following paragraph).

#### Evaluation of peripheral and spinal excitability by femoral nerve stimulation

The femoral nerve stimulation was applied to assess peripheral and spinal excitability. A constant-current and high voltage stimulator (DS7AH, Digitimer, Hertforshire, UK) was used. Rectangular monophasic pulses of 500 μs were used to ensure optimal activation of deeper muscle fibers [38] and to enable the appearance of H-waves [39]. The anode, a self-adhesive electrode (10 × 5 cm), was placed over the greater trochanter. The cathode, a ball electrode covered with damp foam, was placed over the participant’s femoral triangle (Scarpa), 3 to 5 cm below the inguinal ligament. To determine optimal location, the cathode was moved by small amounts while delivering pulses at 50 mA until the highest M-wave response was obtained over the vastus medialis with the smallest possible response over the antagonist biceps femoris. Then markers were set over the participant to maintain the cathode position. A recruitment curve was performed at rest to determine the intensities at which the highest M-wave (Mmax) and H-reflex (Hmax) were obtained. One pulse was delivered on the femoral nerve every 10 s, with the intensity beginning at 50 mA and increasing by 10 mA until no further increase in twitch mechanical response and M-wave amplitude occurred. The intensity used during the protocol was set as 10% above the intensity at which Mmax was elicited (supramaximal intensity noted I_Mmax_). I_Mmax_ was used to evoke M-wave at rest (Mmax) and during maximal voluntary contraction to deliver double twitch pulses (doublet) at 100 Hz. Subsequently to the I_Mmax_ determination, the intensity at which the maximum Hmax was obtained was carefully sought. This intensity was used to evoke H-reflex at rest (Hmax). Peripheral and spinal excitability were defined as the highest Mmax and Hmax recorded during the protocol, respectively. Hmax was normalized with respect to Mmax (Hmax/Mmax) to avoid potential bias due to peripheral excitability differences. Mmax and Hmax latencies were defined as the time between the stimulation onset and the evoked potential onset.

#### Evaluation of corticospinal excitability by transcranial magnetic stimulation

Single transcranial magnetic stimulation (TMS) pulses of 1-ms duration were delivered over the motor cortex using a Magstim 200 (Magstim Co., Whitland, UK). During the settings, TMS pulses were delivered during isometric submaximal voluntary contraction at 10% of the maximal quadriceps torque (facilitation). The figure-of-eight coil was held over the contralateral motor cortex at the optimum scalp position to elicit motor evoked potential (MEP) responses in the contralateral vastus medialis muscle. The contralateral motor cortex was first localized using the 10–10 EEG system (C3 point for right limb stimulation, C4 point for left limb stimulation). Then, the coil was moved by small amounts until the highest MEP response on the vastus medialis was obtained with suprathreshold stimuli, with the smallest possible response over the antagonist biceps femoris, in order to determine the optimal coil location. If significant activation of the antagonist biceps femoris muscle was noted, the coil was slightly moved, until its activation was minimized. Then markers were positioned over the participant and over the coil to maintain the coil location. After that, a recruitment curve was performed during voluntary contraction, at 10% of the maximal quadriceps torque, in order to determine the maximal intensity (noted I_Mep_) [40]. One pulse was delivered every 10 s with increasing intensity in steps of 2% until the highest response was obtained. At least three pulses were delivered at each intensity level to check for reproducibility. The maximal intensity was defined as the intensity at which the highest MEP amplitude was obtained over the vastus medialis. This was then used during the protocol to elicit MEP responses during maximal voluntary contractions in order to assess corticospinal excitability and primary motor cortex activation. If a participant reached the maximum stimulator output without evidence of maximal MEP response (i.e., no evidence of plateau of the MEP amplitude before reaching the maximal output), the data were excluded from the analyses.

Corticospinal excitability was assessed during maximal voluntary contractions by the highest amplitude of the MEP induced by I_MEP_ with respect to peripheral excitability (MEP/Mmax). The silent period duration was measured as the time between the MEP onset and the return of voluntary EMG activity. The central motor conduction time was calculated from the delay between stimulus artifact and the MEP onset.

#### Evaluation of voluntary activation with femoral nerve and transcranial magnetic stimulation

The voluntary activation was assessed by peripheral nerve stimulation (VA_peripheral_) and transcranial magnetic stimulation (VA_cortical_).

VA_peripheral_ was calculated according to the twitch interpolation technique (4). A supramaximal doublet was delivered during the force plateau of the maximal voluntary contraction (superimposed doublet) and 2 s after relaxation (control doublet). VA_peripheral_ was calculated as the ratio between the twitch-like increment in torque induced by the supramaximal doublet during maximal voluntary contraction and after relaxation:

$$ {\mathrm{VA}}_{\mathrm{cortical}}\left(\%\right)=\left[1-\left(\mathrm{superimposed}\ \mathrm{twitch}/\mathrm{estimated}\ \mathrm{resting}\ \mathrm{twitch}\right)\right]\times 100 $$

VA_cortical_ was calculated by stimulating the motor cortex during the quadriceps contractions according to the method described by Sidhu et al. [19]. The estimated resting twitch was calculated from the curve-response relationship obtained by plotting the twitch-like increment in torque induced by the transcranial magnetic pulses delivered during the last two maximal voluntary contractions, as well as those obtained during submaximal voluntary contractions at 30 and 50% of QMVC. When no linear relationship could be obtained between the voluntary force and the twitch-like increment in torque (r < 0.9), the data were excluded from the analyses [41]. VA_cortical_ was calculated as the ratio between the highest twitch-like increment in torque induced by the TMS pulses during maximal voluntary contractions and the estimated resting twitch:

$$ {\mathrm{VA}}_{\mathrm{cortical}}\ \left(\%\right)=\left[1-\left(\mathrm{superimposed}\ \mathrm{twitch}/\mathrm{estimated}\ \mathrm{resting}\ \mathrm{twitch}\right)\right]\times 100 $$

#### EMG activity

The surface EMG activity of the vastus medialis, rectus femoris and biceps femoris was recorded throughout the protocol with Biopac technology (Biopac MP100, Biopac Systems, Santa Barbara, CA, USA). Bipolar, silver chloride, square surface electrodes with a 9-mm diameter were used (Contrôle Graphique Médical, Brie-Compte-Robert, France). In order to minimize impedance (< 5 kΩ), the skin was shaved, abraded, and cleaned with alcohol. Two electrodes were set at the middle belly of the vastus medialis, rectus femoris and long head of the biceps femoris muscles of the dominant leg with an interelectrode distance of 2 cm. The reference electrode was placed on the opposite patella. The EMG signal was band-pass-filtered (10–500 Hz), amplified (× 1000) and recorded at a sample frequency of 4096 Hz.

### Protocol

**Fig. 2:**
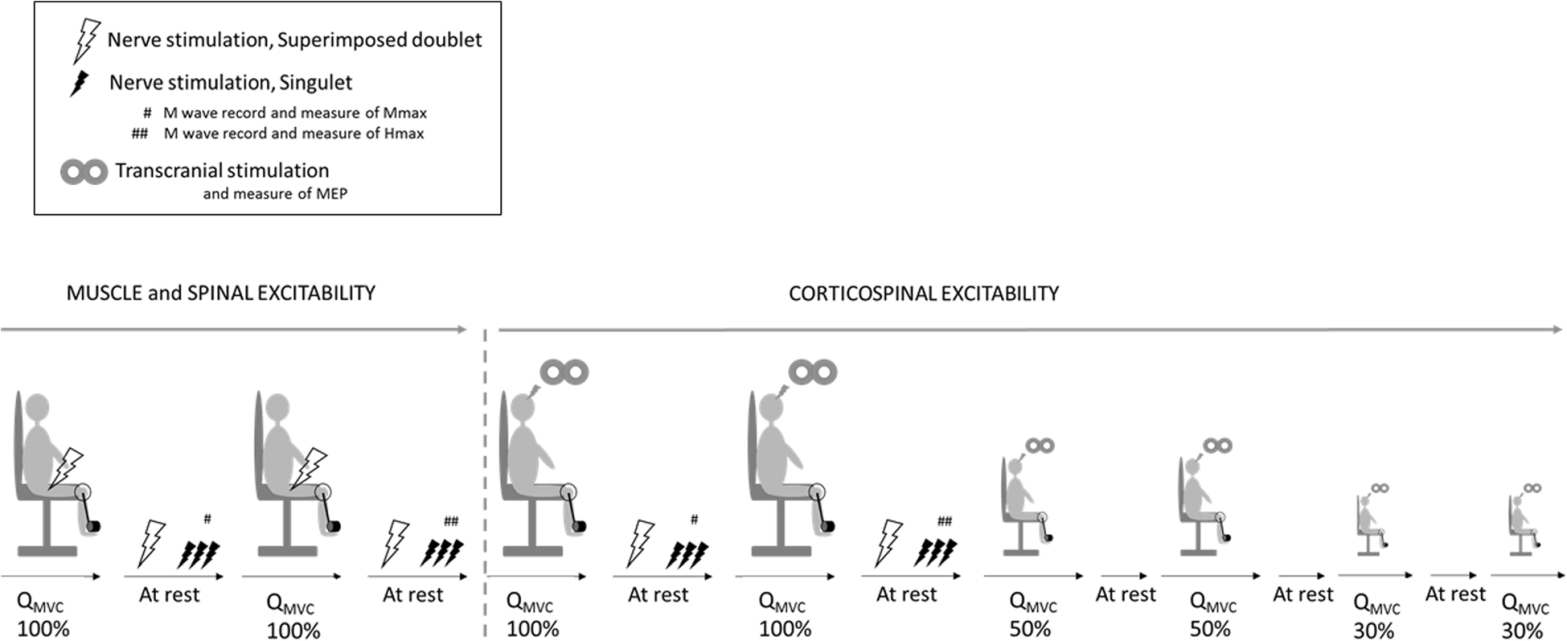
Experimental design. QMVC: Quadriceps voluntary contractions at maximal (100% of QMVC) or submaximal (50 and 30% of QMVC) intensity. Superimposed and control doublets, maximal M-waves (Mmax), and maximal H-waves (Hmax) were delivered via electrical stimulation over the femoral nerve. Motor evoked potentials (MEP) were delivered over the motor cortex via transcranial magnetic stimulation

The participants performed four maximal voluntary contractions of the knee extensors, each separated by 2 min of recovery (Fig. 2). They were asked to maintain maximal effort for at least 4 s. During the first two maximal voluntary contraction maneuvers, a double pulse at 100 Hz was delivered over the femoral nerve (superimposed doublet) during the force plateau and 2 s after relaxation (control doublet). During the last two maximal voluntary contraction maneuvers, a single TMS pulse at I_Mep_ was delivered over the motor cortex to elicit MEPs during the force plateau. Three single pulses at I_Mmax_ or Hmax intensity separated by 10 s were delivered twice between maximal voluntary contractions to elicit Mmax and Hmax at rest, respectively. The time interval between Mmax and Hmax stimuli was between 30 and 40s. If any pre-stimulus voluntary activity was observed, the involved stimuli were discarded. After the maximal voluntary contractions, three submaximal voluntary contractions (SVC) with visual feedback were performed at 50 and 30% of QMVC. A single TMS pulse at I_Mep_ was delivered during the force plateau of each SVC to elicit superimposed twitch responses at 30 and 50% of QMVC.

### Statistical analyses

All statistical analyses except slope comparisons were performed using Statistica software (StatSoft, Inc., version 6.0, Tulsa, OK, USA). All data were examined for normality using a Shapiro-Wilk test. Differences between the pooled COPD patients and the healthy controls were studied using unpaired t-tests for parametric data and the non-parametric Mann-Whitney U test otherwise. Differences between the COPD_MW_ and COPD_NoMW_ groups and healthy controls were tested using a one-way between-subject analysis of variance (ANOVA), unless when no data were available for healthy controls (i.e. blood gas analyses and comorbidities), with the 2 groups of patients compared using unpaired t-test instead. The underlying assumptions of ANOVA were checked using a Levene test (homogeneity of the variance). When the ANOVA F ratio was significant (*p* < 0.05), the means were compared by a Studentized Newman-Keuls (SNK) post-hoc test. Analysis of covariance (ANCOVA) was used with 1) QMVC as the criterion variable and QPt as the covariate (adjusted maximal voluntary strength), 2) VA_cortical_ as the criterion variable and PaO_2_ as the covariate. Bivariate regression analyses were performed using the Pearson coefficient. The slopes and Y-intercepts of the relationships between QMVC and QPt were compared for differences between the three groups using a specific ANOVA procedure of the Statgraphics Centurion XVII statistical package. Data are presented mean ± standard error (SE).

## Results

### Controls and the pooled COPD patients

The results from the COPD patients taken as a whole and the controls are indicative and are provided in Table 1. COPD patients presented significantly lower QMVC (t_58_ = 3.42, *p* < 0.05) and QPt (t_57_ = 2.31, *p* < 0.05) than controls. Moreover, they also exhibited significantly higher Hmax latency (t_37_ = 2.94, *p* < 0.01) and longer silent period duration (t_37_ = 3.33, *p* < 0.001) than controls. Interestingly, there were no significant differences between the patients and controls regarding VA_cortical_ and MEP/Mmax (*p* = 0.26 and 0.68, respectively).

**Table 1:** Comparison between healthy controls and patients with COPD as a whole

	Controls *n* = 20	COPD *n* = 40	*p*-value (*t*-test)
Anthropometric parameters			
Gender, M/F	10/10	21/19	–
Age, yrs	61.3 (2.17)	61.7 (1.73)	0.97
Weight, kg	72 (2.59)	73 (3.96)	0.89
BMI, kg.m^−2^	25.4 (0.76)	26.4 (1.26)	0.46
Pulmonary parameters			
FEV_1_, L	3.12 (0.2)	1.3 (0.11)	< 0.001
FEV_1_, % pred	113.9 (3.2)	49.6 (3.66)	< 0.001
FEV_1_/FVC	0.74 (0.46)	0.5 (0.28)	< 0.001
Maximal voluntary and electrically evoked quadriceps torque			
QMVC, Nm	150.3 (15.7)	99 (10.4)	< 0.05
QPt, Nm	43.1 (3.71)	33.5 (3.32)	< 0.05
Voluntary activation parameters			
VA_peripheral_	93 (1)	93.1 (1.32)	0.94
VA_cortical_, %	89.8 (1.02)	85.7 (3.11)	0.26
Peripheral and spinal parameters			
Mmax amplitude, mV	3.94 (0.38)	3.32 (0.5)	0.36
Mmax lat, ms	5.7 (0.27)	6.15 (0.25)	0.38
Hmax/Mmax	0.188 (0.027)	0.26 (0.029)	0.34
Hmax lat, ms	16.63 (0.54)	18.98 (0.59)	< 0.01
Corticospinal parameters			
MEP/Mmax	0.374 (0.021)	0.34 (0.045)	0.68
CMCT, ms	20.3 (0.56)	20.2 (0.54)	0.59
CSP, ms	75.6 (7.2)	103 (4.43)	< 0.001

*BMI* body mass index, *FEV*_*1*_ forced expiratory volume in 1 s, *FVC* forced vital capacity, *QMVC* quadriceps maximal voluntary torque, *QPt* quadriceps peak twitch, *Cortical VA* primary motor cortex activation, *Mmax amplitude* amplitude of the maximal M-wave, *Mmax lat* latency of the maximal M-wave, *Hmax/Mmax* amplitude of the maximal H-wave normalized with respect to the maximal M-wave, *Hmax - Mmax lat* latency of the maximal H-wave minus the latency of the maximal M-wave, *MEP/Mmax* amplitude of the maximal motor evoked potential normalized with respect to the maximal M-wave amplitude, *CMCT* central motor conduction time, *CSP* cortical silent period. Data are expressed as mean and SE

### Controls and the COPD_MW_ and COPD_NoMW_ groups

#### Participant characteristics

The participant characteristics are presented in Table 2. The COPD_MW_ and COPD_NoMW_ groups were composed of 22 (M/F ratio = 10/12) and 18 patients (M/F = 11/7), respectively. There were no significant differences between the three groups in age, weight or BMI (*p*-value equal to 0.84, 0.67 and 0.53, respectively). The two groups of COPD patients did not differ regarding airflow obstruction (FEV_1_/FVC), obstruction severity (FEV_1_), smoking pack-years and number of comorbidities (*p* = 0.48, 0.62, 0.39 and 0.63, respectively). In contrast, PaO_2_ was significantly lower in the COPD_MW_ group (*p* < 0.01).

**Table 2:** Characteristics of controls and patients with (COPD_MW_) or without (COPD_NoMW_) muscle weakness

	Controls (a)	COPD_NoMW_ (b)	COPD_MW_ (c)	ANOVA *p*-value	*p*-value a vs b	*p*-value a vs c	*p*-value b vs c
Gender, M/F	10/10	11/7	10/12	–	–	–	–
Age, yrs	61.3 (2.17)	60.8 (2.19)	62.4 (1.34)	0.84	–	–	–
Weight, kg	72 (2.59)	75.3 (3.42)	71.1 (3.94)	0.67	–	–	–
BMI, kg.m^−2^	25.4 (0.76)	27.3 (1.18)	25.8 (1.41)	0.53	–	–	–
FEV_1_, L	3.12 (0.2)	1.34 (0.14)	1.23 (0.09)	< 0.001	< 0.001	< 0.001	0.62
FEV_1_, % pred	113.9 (3.2)	50.5 (4.21)	47.4 (3.18)	< 0.001	< 0.001	< 0.001	0.53
FEV_1_/FVC	0.74 (0.11)	0.51 (0.32)	0.49 (0.27)	< 0.001	< 0.001	< 0.001	0.53
PaO_2_, mmHg	–	72.2 (2.07)	65.4 (1.55)	–	–	–	< 0.01*
PaCO_2_, mmHg	–	40.1 (1.59)	40.1 (1.1)	–	–	–	0.99*
Smoking history (pack-years)	7.3 (12.5)	38.9 (26.9)	45.5 (24)	< 0.001	< 0.001	< 0.001	0.39
Number of comorbidities	–	2 (1.2)	2.2 (1.1)	–	–	–	0.63*

*BMI* Body mass index, *FEV*_*1*_ forced expiratory volume in 1 s, *FVC* forced vital capacity, *PaO*_*2*_ arterial oxygen tension, *PaCO*_*2*_ arterial carbon dioxide tension. Data are mean and SE

#### Maximal voluntary and electrically evoked quadriceps torque

**Fig. 3:**
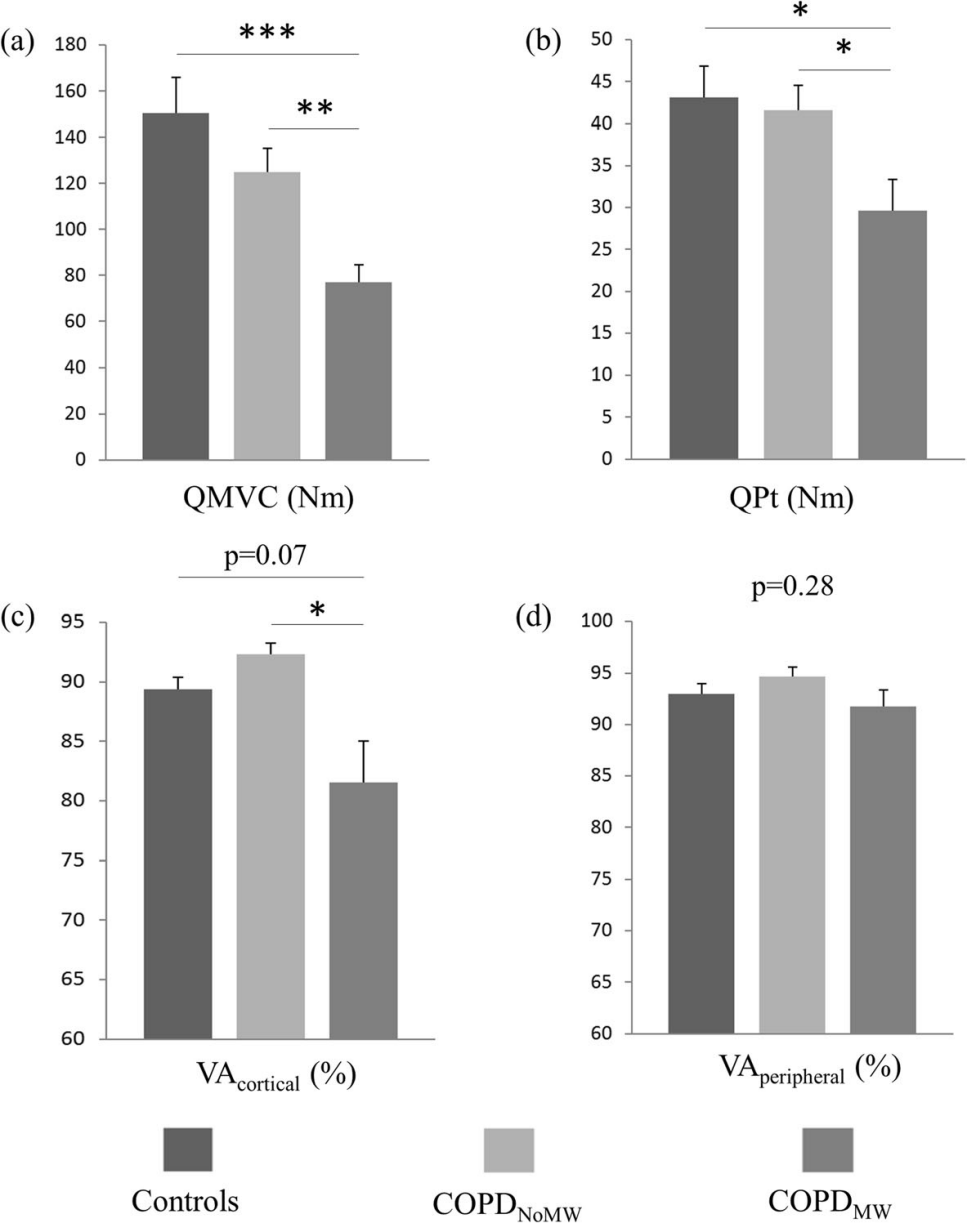
**a** Quadriceps maximal voluntary torque (QMVC), **b** quadriceps maximal peak twitch (QPt), **c** voluntary activation estimated with motor cortex transcranial magnetic stimulation during maximal voluntary contractions [VA_cortical_ (%)], and **d** voluntary activation estimated with twitch interpolation during maximal voluntary contractions [VA_peripheral_ (%)], in healthy controls, patients with COPD and preserved muscle strength (COPD_NoMW_), and patients with COPD and muscle weakness (COPD_MW_). *, **, ***: *p* < 0.05, *p* < 0.01 and *p* < 0.001 respectively. Vertical bars represent SE

The data are presented in Fig. 3a and b. Significant effects of group were found for QMVC and QPt (F_2,57_ = 10.73, *p* < 0.001 and F_2,57_ = 4.46, *p* < 0.01, respectively), which were significantly lower in COPD_MW_ compared with the COPD_NoMW_ and control groups (all post-hoc *p* < 0.05). Even adjusted for QPt, QMVC remained significantly lower in COPD_MW_ (significant effect of group: F_2,56_ = 6.54, *p* < 0.01; with both post-hoc *p* < 0.05). There was no difference in QMVC or QPt between the COPD_NoMW_ and control groups (post-hoc *p* = 0.14 and 0.77, respectively). In addition, the percentage of QMVC variance explained by QPt was quite different between healthy controls (adjusted r^2^ = 0.8) and COPD patients (adjusted r^2^ = 0.65 and 0.62 for COPD without and with muscle weakness, respectively).

**Fig. 4:**
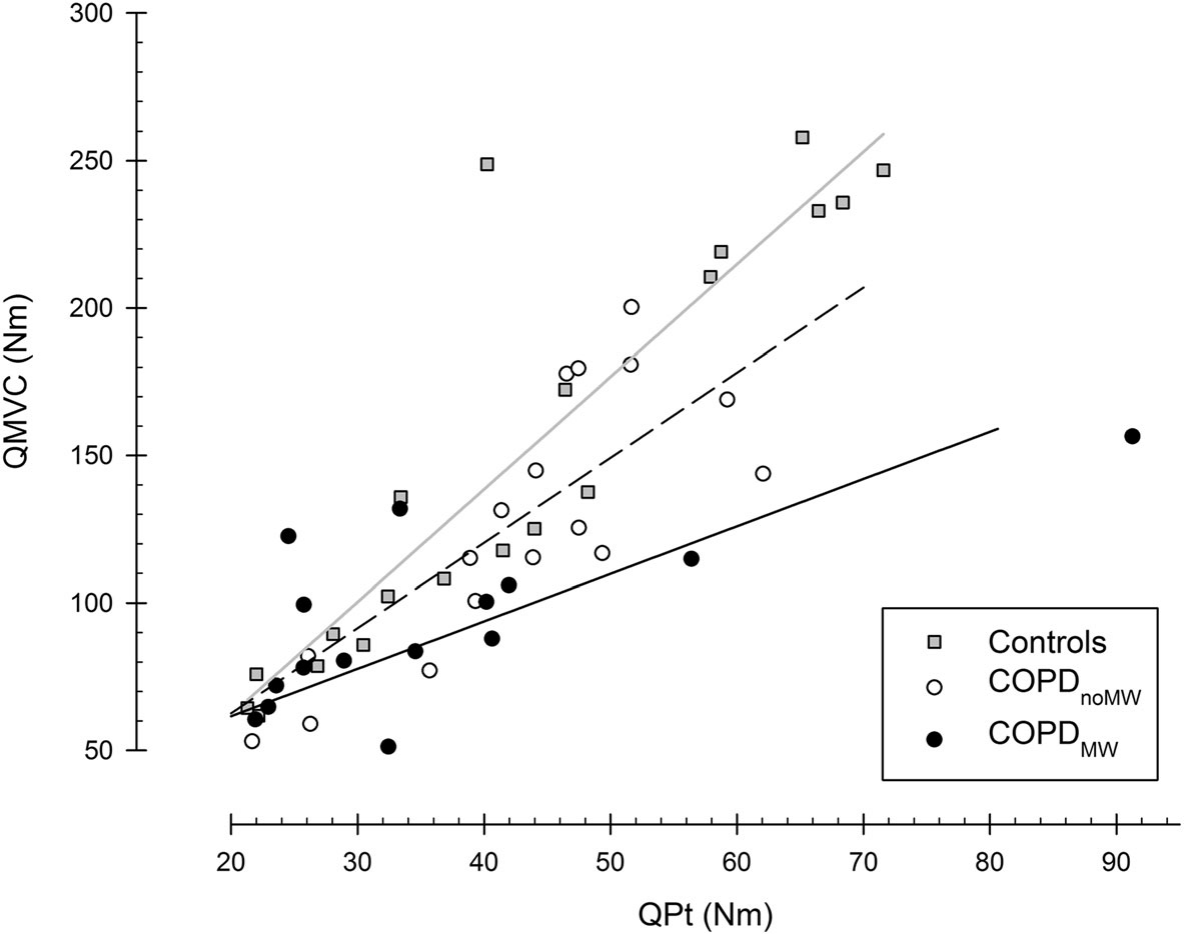
Slopes and y-intercepts of linear relationship between maximal voluntary quadriceps torque (QMVC) and quadriceps maximal peak twitch (QPt) in healthy controls (gray squares and gray line), COPD patients without muscle weakness (COPD_NoMW_) (empty circles and dotted line), and COPD patients with muscle weakness (COPD_MW_) (dark circles and dark line)

Correlations between QMVC and QPt were statistically significant in all groups (r ranged from 0.8 to 0.9; p systematically lower than 0.001). However, the characteristics of the relationship (slopes and y-intercepts) differed according to the group. The y-intercept was progressively higher in the COPD_NoMW_ (4.8) and COPD_MW_ (29.4) groups compared with the control group (− 14) (F_5,54_ ranged from 4.53 to 18.9, p ranged from 0.001 to 0.04). The slope was not different between the control (3.82) and COPD_NoMW_ (2.8) groups (F_3,34_ = 1.82; *p* = 0.18) but was significantly lower in the COPD_MW_ group (1.6) (comparison versus COPD_NoMW_: F_3,36_ = 5.25; *p* < 0.03 and versus controls: F_3,38_ = 20.4; *p* < 0.001; see Fig. 4). As a consequence, for equivalent QPt, QMVC tended to be lower in the COPD_MW_ group.

#### Peripheral and corticospinal excitability

The electrophysiological data are presented in Table 3. Sixteen participants (26.6%: 5 controls, 5 COPD patients with muscle weakness, and 6 COPD patients without muscle weakness) did not exhibit maximal MEP at maximal stimulator output or had no linear relationship between TMS twitches. These participants were excluded from the analyses of data elicited by TMS. Muscular excitability estimated by Mmax amplitude and Mmax latency did not significantly differ between the 3 groups (F_2,54_ = 1.03, *p* = 0.36 and F_2,56_ = 0.97, *p* = 0.38, respectively). Spinal excitability expressed by the Hmax/Mmax ratio was also comparable between the 3 groups (F_2,35_ = 1.44, *p* = 0.25). A significant effect of group was found for Hmax latency (F_2,36_ = 4.26, *p* < 0.05), which was significantly higher in the two COPD groups compared with controls (both post-hoc *p* < 0.05). However, the central motor conduction time was not different between the three groups (F_2,35_ = 1.16, *p* = 0.33). A significant effect of group was found for corticospinal excitability measured by MEP/Mmax ratio (F_2,33_ = 7.19, *p* < 0.01), which was significantly lower in the COPD_MW_ group compared with the COPD_NoMW_ and control groups (post-hoc *p* < 0.05 and 0.01, respectively). There was also a significant effect of group for the silent period duration, which is an indicator of corticospinal inhibition (F_2,36_ = 6.07, *p* < 0.01). Post-hoc comparisons revealed that the silent period duration was longer in the two COPD groups compared with controls (*p* < 0.05 for comparison between COPD_NoMW_ and controls, and *p* < 0.01 for COPD_MW_ and controls), without any differences between the two COPD groups (*p* = 0.31).

**Table 3:** Muscle and corticospinal excitability assessment

	Controls (a)	COPD_NoMW_ (b)	COPD_MW_ (c)	ANOVA *p*-value	*p*-value a vs b	*p*-value a vs c	*p*-value b vs c
Muscle and spinal parameters							
Mmax amplitude, mV	3.94 (0.38)	2.96 (0.3)	3.58 (0.55)	0.36	–	–	–
Mmax lat, ms	5.7 (0.27)	6.21 (0.26)	6.11 (0.27)	0.38	–	–	–
Hmax/Mmax	0.188 (0.027)	0.25 (0.041)	0.265 (0.02)	0.25	–	–	–
Hmax lat, ms	16.63 (0.54)	18.86 (0.62)	19.17 (0.51)	< 0.05	< 0.05	< 0.05	0.77
Corticospinal parameters							
MEP/Mmax	0.374 (0.021)	0.464 (0.056)	0.235 (0.019)	< 0.01	0.22	< 0.01	< 0.05
CMCT, ms	20.3 (0.56)	19.3 (0.45)	20.8 (0.55)	0.33	–	–	–
SP, ms	75.6 (7.2)	108.3 (4.55)	98.3 (3.79)	< 0.01	< 0.01	< 0.05	0.31

*Mmax amplitude* amplitude of the maximal M-wave, *Mmax lat* latency of the maximal M-wave, *Hmax/Mmax* amplitude of the maximal H-wave normalized with respect to the maximal M-wave, *Hmax lat* latency of the maximal H-wave minus the latency of the maximal M-wave, *MEP/Mmax* amplitude of the maximal motor evoked potential normalized with respect to the maximal M-wave amplitude, *CMCT* central motor conduction time, *SP* silent period duration. Data are mean and SE

#### Voluntary activation

The mean correlation coefficient of the relationship between twitch-like increment in torque induced by TMS and voluntary torque was 0.979 ± 0.03. A significant effect of group was found for VA_cortical_ (F_2,39_ = 3.91, *p* < 0.05; Fig. 3c) which was significantly lower in COPD_MW_ compared with COPD_NoMW_ (post hoc *p* < 0.01). Even adjusted for PaO_2_, VA_cortical_ remained significantly lower in COPD_MW_ (F_2,38_ = 5.87, *p* < 0.05).

Conversely, VA_peripheral_ (Fig. 3d) did not differ between groups (F_2,57_ = 1.32, *p* = 0.28).

## Discussion

The purpose of the study was to compare corticospinal and muscle functions in COPD patients with and without peripheral muscle weakness. Compared with patients with preserved muscle strength, patients with muscle weakness exhibited lower VA_cortical_ during maximal voluntary contractions associated with a lower MEP/Mmax ratio. Conversely, no differences were found between patients with and without muscle weakness regarding silent period duration, central motor conduction time or M-wave properties.

Peripheral muscle alterations were assessed by QPt (non-voluntary contraction, reflecting both muscle structure and function) in this study. We found lower QPt in patients with muscle weakness compared with healthy controls and patients with normal muscle strength. These findings concur with those of a previous study highlighting the implication of peripheral alterations in the poor quadriceps strength of the weakest COPD patients [21]. However, several clues are in agreement with a moderate role. First, when QPt was included as a covariate in ANCOVA analysis, the maximal voluntary contraction (QMVC) remained significantly lower in COPD_MW_. Second, the percentage of QMVC variance explained by QPt was sharply lower in COPD patients with muscle weakness (62%) than in control subjects (80%). These data indicate that peripheral muscle alterations are less decisive in maximal force production in COPD patients with muscle weakness. Third, as shown in Fig. 4, the slope between QMVC and QPt was significantly lower in patients with muscle weakness compared with patients without muscle weakness and healthy controls. In other words, for a given QPt, the patients with muscle weakness tended to exhibit lower QMVC than the other groups. Collectively, these data point to the existence of an additional non-muscular limiting factor in COPD muscle weakness. The reduced neural excitability and the voluntary activation deficit that we found in parallel in the patients with muscle weakness are relevant explanations for the unexplained part of COPD muscle weakness.

The existence of a voluntary activation deficit has been a controversial issue in COPD. A first consideration is the relevance of the twitch interpolation method during maximal voluntary contractions to assess voluntary activation [11–14]. In the current study, we provided an additional estimation of voluntary activation by directly stimulating at the motor cortex level. We observed lower VA_cortical_ in the patients with muscle weakness, despite no VA_peripheral_ differences. This is not the first time that a mismatch between VA_cortical_ and VA_peripheral_ has been reported over the knee extensor muscles [42–44]. The absence or relatively poor VA_peripheral_ changes compared with the VA_cortical_ changes has been explained by a lack of sensitivity of the twitch interpolation method [42, 44]. Indeed, this method cannot provide a direct indication of the amount of neural drive reaching the muscle because the assessment takes place at the muscle level [45, 46]. Moreover, the twitch interpolation method presents a nonlinear relationship at high force levels, such that changes in the voluntary force elicit minimal changes in the superimposed twitch size [16–18]. For example, in a previous study, a gain of 5.7% in VA_peripheral_ induced an average 20.4% increase in QMVC [18]. Another consideration is that voluntary activation and neural activity have previously been assessed in COPD patients as a whole without discriminating the patients with and without muscle weakness [11–15, 47]. At first glance, the absence of a significant difference in VA_cortical_ between the controls and the patients as a whole (Table 1) might have led us to conclude that an activation deficit was probably not involved in the reduced quadriceps strength in COPD. However, the patients with quadriceps muscle weakness exhibited a significantly lower VA_cortical_ compared with patients with preserved quadriceps strength. This result suggests that in many patients, reduced motor cortex activation is involved in quadriceps muscle weakness. A lower MEP/Mmax ratio was also noted in the patients with quadriceps muscle weakness. Changes in MEP/Mmax can reflect changes in spinal or cortical excitability [48]. In the current study, spinal excitability (Hmax/Mmax) did not differ between groups. Thus, the lower MEP/Mmax ratio suggests reduced motor cortex excitability in the patients with quadriceps muscle weakness, which supports the hypothesis of reduced voluntary activation from the motor cortex in these patients. Importantly, the only difference in pulmonary and blood gas data between the two groups of patients concerned the resting PaO_2_ levels, which were significantly lower in the weak patients. To check that the VA_cortical_ alteration was not biased by hypoxemia differences, we performed an ANCOVA with PaO_2_ as covariate. VA_cortical_ remained significantly lower in the patients with muscle weakness (F = 5.87, *p* < 0.05). Furthermore, any oxygen desaturation during the quadriceps contraction of the study protocol was unlikely, since in a previous study where SpO2 was measured during similar efforts, the mean variation of SpO_2_ was only 0.01 and non-significant [15]. Consequently, the significantly lower VA_cortical_ in the weak patients can be considered as a consistent result.

In this study, we also aimed to assess the mechanisms potentially involved in the decreased voluntary activation in COPD. Silent period duration is thought to reflect the level of corticospinal inhibition [49]. In accordance with a previous study, we found a lengthened silent period in COPD patients compared with healthy controls [50]. However, the increased silent period duration in both groups of COPD patients regardless of muscle weakness necessarily indicates that higher corticospinal inhibition is not responsible for most of the observed loss of voluntary strength in the patients with muscle weakness.

Peripheral neuropathy has been widely described in COPD and has mainly been characterized by lower peripheral nerve conduction velocities and lower Mmax amplitude [25–28]. In the current study, we found no differences regarding Mmax amplitude and latency between the two groups of patients and the healthy controls. These results suggest the preservation of neuromuscular transmission at the motor neuron and/or the motor plate level in COPD. This is also supported by the comparable central motor conduction time (corresponding to the MEP latency) between groups, which depends on both motor neuron and corticospinal conduction velocities. Conversely, we noted lengthened spinal reflex latency (Hmax latency) in the patients with COPD compared with healthy controls, regardless of muscle weakness. The alteration in Hmax latency without any central motor conduction time changes could be explained by impairment at the Ia afferent pathways, as the other pathways traveled by the H-reflex are the same as those traveled by the MEP. Therefore, these results suggest a selective alteration in the quadriceps Ia afferent pathways in the patients with COPD, which is in agreement with previous studies reporting greater impairment in the fascicles of sensory nerves than in motor nerves in these patients [25]. Moreover, the lengthened Hmax latency regardless of muscle weakness also indicates that the alterations in the sensory nerve fascicles are not responsible for most of the observed loss of voluntary strength in the patients with muscle weakness.

In sum, higher corticospinal inhibition and impaired neuromuscular transmission are unlikely to be involved in the reduced quadriceps strength of the patients with muscle weakness. The most likely explanatory mechanism of the neural component of peripheral muscle weakness, and thus for reduced voluntary activation, is decreased excitation from the brain, which is supported by the observation of lower gray matter density in the motor cortex (precentral gyrus) in COPD [22].

Although not a direct objective of the current study, our results may provide some new insights in the potential trigger for brain impairment in COPD. Several etiological factors have been advanced to explain brain impairment in COPD, the most important of which are cerebral vascular disease, inflammation, oxidative stress, smoking, hypoxemia and non-rapid eye movement (NREM) sleep desaturation [51, 52]. First, we did not find any differences in smoking history between the two groups of COPD patients. Thus, a major implication of cigarette smoke in the reported brain alterations is unlikely. Furthermore, without being able to definitely discard the implication of hypoxemia, our results show that the reduced cortical activation was independent of PaO_2_ levels.

Beyond mechanistic perspectives, the results of the study open new horizons for muscle weakness management in patients with COPD. Indeed, some specific interventions are known to promote neural adaptations such as eccentric exercise [53], neuromodulation [54] or electrical stimulation strength training [55]. These interventions might help to improve responses to rehabilitation in COPD patients with muscle weakness from cortical origin. This is particularly relevant for efforts to improve adaptations to pulmonary rehabilitation programs, given the rate of patients who respond poorly to the classical programs [56].

Methodological considerations.

We used a figure-of-eight coil to stimulate the leg motor cortex. Although a double-cone coil is more conventional to stimulate the leg motor cortex as the magnetic stimulus penetrates less deeply for a given intensity [57], the figure-of-eight coil takes the advantage to be more focal [58], and there is no clear rationale to give priority to one over the other in the literature [57]. Apart from these considerations, some strict procedures helped us to increase the validity of the data provided by the figure-of-eight coil. First, to avoid potentially suboptimal output, we used a higher intensity (mean stimulator output was 96%) than the intensities usually reported with a double-cone coil (≈60–70%). We also excluded from the TMS data analyses the participants with no MEP plateau before reaching the maximal stimulator output (26.6%). This rate of exclusion was not much higher than in previous studies. For example, in younger healthy subjects, a rate of exclusion between 15 and 20% was reported [59, 60]. In addition, the TMS voluntary activation data were quite consistent with the literature data. In the current study, the mean TMS voluntary activation using a figure-of-eight coil was 89% in the healthy elderly controls and 92% in the patients without muscle weakness. In a previous study using a double-cone coil on the same muscle group and on a similar population (healthy elderly), mean TMS voluntary activation was 90% [61].

Another limitation was that the antagonist biceps femoris Mmax could not be measured in the study. The minimization of antagonist muscle activity during TMS is important to provide appropriate TMS data [41]. However, the stimulation of the sciatic nerve to elicit biceps femoris Mmax is very challenging for technical and ethical reasons. First, the site of stimulation is surrounded by large muscles, which makes it difficult to avoid stimulating a muscle directly. Furthermore, to evoke Mmax on the biceps femoris, a very high stimulation intensity on the sciatic nerve (over 600 mA) is needed [19]. This intensity is more than six times higher than that used for stimulating the femoral nerve in the current study. The use of such painful intensity is inconceivable in patients with chronic illness. Nevertheless, several strategies were employed to minimize antagonist activity during TMS. First, the skin impedance was rather null in both groups (< 5 kΩ). Second, we systematically checked to obtain the smallest possible response on the antagonist muscle by moving the coil by small amounts. Last, the study included a control group and, given the two aforementioned procedures, there is no reason to believe that the antagonist was more activated in one group than the others.

## Conclusions

In addition to peripheral muscle alterations (lower QPt), patients with COPD and muscle weakness exhibit lower motor cortex excitability during maximal voluntary contractions and, logically, lower voluntary activation from the motor cortex. A decrease in neural excitation is the most likely mechanism responsible for the lower motor cortex activation in these patients. This study provides evidence of specific cortical implication in COPD with muscle weakness. This may have important implications for pulmonary rehabilitation and it could potentially explain why some patients exhibit low responsiveness to retraining programs. Thus, the question of specific interventions in patients with muscle weakness of cortical origin needs to be addressed. Further, future studies and research must be carried out on the factors responsible for motor cortex impairment in COPD and the possible therapeutic targets.

## Data Availability

The datasets that were used and analyzed during the current study are available from the corresponding author on reasonable request.

## Abbreviations

ANCOVA: Analysis of covariance
ANOVA: Analysis of variance
COPD: Chronic obstructive pulmonary disease
COPD_MW_: COPD patients with quadriceps muscle weakness
COPD_noMW_: COPD patients with quadriceps strenght
EMG: Electromyography
FEV_1_: Forced expiratory volume in the 1st second
FVC: Forced vital capacity
Hmax: Maximal H-reflex
I_Mmax_: Intensity at which Mmax is obtained
MEP: Motor evoked potential
Mmax: Maximal compound muscle action potential
QMVC: Isometric maximal quadriceps torque
QPt: Quadriceps Peak twitch
TMS: Transcranial magnetic stimulation
VA_cortical_: Voluntary activation estimated by TMS
VA_peripheral_: Voluntary activation estimated by peripheral nerve stimulation
